# The efficacy of molecular targeted therapy and nivolumab therapy for metastatic non‐clear cell renal cell carcinoma: A retrospective analysis using the Michinoku Japan urological cancer study group database

**DOI:** 10.1002/cam4.6591

**Published:** 2023-10-31

**Authors:** Tomoyuki Koguchi, Sei Naito, Shingo Hatakeyama, Kazuyuki Numakura, Yumina Muto, Renpei Kato, Takahiro Kojima, Yoshihide Kawasaki, Kento Morozumi, Shuya Kandori, Sadafumi Kawamura, Hiroyuki Nishiyama, Akihiro Ito, Tomonori Habuchi, Wataru Obara, Chikara Ohyama, Norihiko Tsuchiya, Yoshiyuki Kojima

**Affiliations:** ^1^ Department of Urology Fukushima Medical University School of Medicine Fukushima Japan; ^2^ Department of Urology Yamagata University Faculty of Medicine Yamagata Japan; ^3^ Department of Urology and Advanced Blood Purification Therapy Hirosaki University Graduate of Medicine Hirosaki Japan; ^4^ Department of Urology Akita University Graduate School of Medicine Akita Japan; ^5^ Department of Urology Iwate Medical University School of Medicine Iwate Japan; ^6^ Department of Urology University of Tsukuba, Graduate School of Comprehensive Human Sciences Tsukuba Japan; ^7^ Department of Urology Tohoku University School of Medicine Sendai Japan; ^8^ Department of Urology Miyagi Cancer Center Natori Japan

**Keywords:** immune checkpoint inhibitor, immune‐oncology, molecular target therapy, non clear cell renal cell carcinoma

## Abstract

**Objectives:**

To investigate the efficacy of pharmacotherapy for metastatic non‐clear cell renal cell carcinoma (nccRCC) in Japanese population.

**Methods:**

In this retrospective analysis, we compared the time to treatment failure (TTF) for molecular‐targeted agents as first‐line therapy, or nivolumab therapy as sequential therapy between ccRCC and nccRCC using the data of Japanese metastatic RCC patients registered in the Michinoku Japan Urological Cancer Study Group database.

**Results:**

In total, 511 cases of ccRCC and 77 cases of nccRCC were treated with pharmacotherapy. After excluding the patients who received cytokine therapy, chemotherapy, or others, there were 391 ccRCC patients and 60 nccRCC patients who were treated with tyrosine kinase inhibitors (TKIs), and 7 ccRCC patients and 7 nccRCC patients who were treated with mammalian‐target of rapamycin inhibitors (mTORIs). In addition, 132 ccRCC patients and 16 nccRCC patients received nivolumab. There was no significant difference in IMDC risk classification before first‐line therapy between ccRCC and nccRCC groups, or in each subgroup within the nccRCC group. TTF for TKIs (161 days, 95% CI: 75‐212 days) and mTORIs (21 days, 95% CI: 9‐31 days) didn’t differ significantly between nccRCC and ccRCC groups (205 days, 95% CI: 174‐243 days and 33 days, 95% CI: 8‐113 days, respectively). TTF for TKIs was significantly longer than that for mTORIs in nccRCC group (p<0.01). There was no significant difference in TTF between the different TKIs in nccRCC group. In addition, no significant difference in TTF for nivolumab was seen between ccRCC and nccRCC groups.

**Conclusions:**

The results showed that the efficacy of molecular‐targeted agents as first‐line therapy was similar oncological outcomes between metastatic nccRCC and ccRCC in Japanese patients. TKIs may be more effective than mTORIs in metastatic nccRCC patients. Nivolumab administration might also be as effective in nccRCC patients as in ccRCC patients in Japanese population.

## 1 INTRODUCTION

Clear cell renal cell carcinoma (ccRCC) accounted for the majority of renal cancers (70%–85%), and most of the patients with metastatic renal cancer also have ccRCC.^1^, ^2^, ^3^, ^4^ Recently, there has been progress in the development of systemic drug therapies such as molecular targeted agents and immune checkpoint inhibitors, for renal cancer, and previous reports have shown that these therapies are useful for treating ccRCC.^5^ However, non‐clear cell renal cell carcinoma (nccRCC) differs from ccRCC in that it comprises various histological types, and there are fewer case numbers. Since few recommended systemic drug therapy protocols for nccRCC; the same drug therapies used for ccRCC has also applied to nccRCC despite that the efficacy of these drug therapies is lower in nccRCC than in ccRCC.^5^ According to previous reports, although sunitinib and temsirolimus are recommended for metastatic nccRCC,^6^, ^7^, ^8^ the evidences were based on studies with only a small number of cases. The accumulation of more data on the efficacy of drug therapies for nccRCC is desirable.^3^, ^9^

Immune checkpoint inhibitors have been reported to be effective in patients with renal cell carcinoma (RCC).^10^ Previous studies have demonstrated the efficacy of nivolumab in metastatic renal cell carcinoma (mRCC), and that nivolumab may yield long‐term survival benefits.^11^, ^12^ However, most of these studies on mRCC were conducted on ccRCC, and few studies have been conducted on nccRCC.^13^

In the present study, to investigate the efficacy of systemic drug therapies for nccRCC, we performed a retrospective analysis and compared the time to treatment failure (TTF) for molecular targeted agents that were administered as first‐line therapy, and the TTF for nivolumab therapy between ccRCC and nccRCC cases using data from the Michinoku Japan Urological Cancer Study Group database as a Japanese metastatic renal cancer database.

## 2 METHODS

### 2.1 Patient characteristics and clinical data

We retrospectively analyzed the data of 703 patients who were diagnosed with mRCC between January 2008 and August 2018 in the Michinoku Japan Urological Cancer Study Group database. The last follow‐up date was November 2019.

**FIGURE 1:**
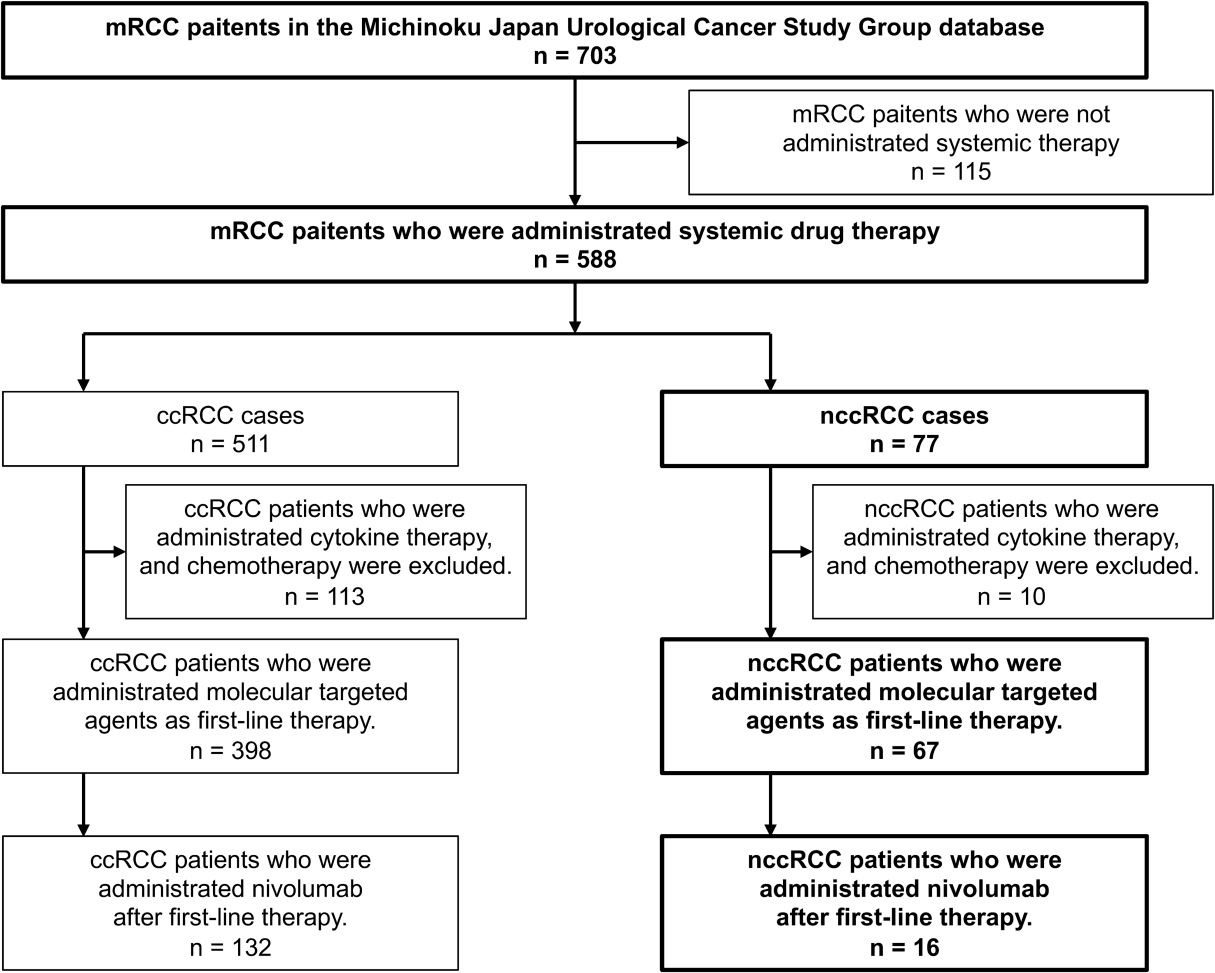
Selection of patients. Of the total 703 patients, we selected 588 patients with primary metastatic renal cell carcinoma who received systemic drug therapy; 511 patients had clear cell renal cell carcinoma (ccRCC), and 77 patients had non‐clear cell renal cell carcinoma (nccRCC). After excluding patients who received cytokine therapy, chemotherapy, or others, there were 398 patients with ccRCC, and 67 patients with nccRCC who received molecular targeted agents as first‐line therapy. In addition, 132 patients with ccRCC, and 16 patients with nccRCC received nivolumab after first‐line therapy.

There were 588 patients with metastatic renal cancer (511 patients with ccRCC, and 77 patients with nccRCC) who received systemic drug therapy. The systemic drug therapies included tyrosine kinase inhibitors (TKIs), mammalian target of rapamycin inhibitors (mTORIs), immune checkpoint inhibitors, cytokine therapy, and chemotherapy. The TKIs were sunitinib, sorafenib, axitinib, and pazopanib. Currently, mRCC cases are treated mainly with molecular targeted therapy and an immune‐oncology (IO) therapy; therefore, patients who received cytokine therapy and chemotherapy, were excluded from the present analysis (Figure 1).

In this study, we evaluated the usefulness of systemic molecular targeted therapies as first‐line therapy. At the time of our study, mRCC cases could not be treated with nivolumab as first‐line therapy in Japan due to the regulations of the national medical insurance system; hence, we analyzed the efficacy of nivolumab administered as sequential therapy.

### 2.2 Ethics

This study was approved by the Ethics Committee of Fukushima Medical University School of Medicine (approval no. 2019–115). The study was conducted in compliance with all appropriate national and international ethical guidelines, and complied with the Act of Protection of Personal Information. The need for consent to participate in this study was waived by the ethics committee.

### 2.3 Data‐analysis items

The International mRCC Database Consortium (IMDC) risk score was determined by the presence of the following risk factors (1 point each); Karnofsky performance status (KPS) < 80%; hemoglobin level below the lower limit of normal; corrected serum calcium level ≥ 10 mg/dL; period from RCC diagnosis to the treatment start date <1 year; neutrophil count at or above the upper limit of normal (≥ ULN); and platelet count ≥ ULN.^14^ The TTF for first‐line therapy was defined as the period from the start date of first‐line therapy administration to the start date of second‐line therapy administration, the date of death, or the data cutoff date.^15^ Similarly, the TTF for nivolumab therapy after prior therapy with systemic molecular targeted therapies was defined as the period from the start date of nivolumab administration to the start date of next‐line therapy administration, the date of death, or the data cutoff date. For the TTF, graphical outputs were created based on the Kaplan–Meier methodology.

### 2.4 Statistical analysis

All statistical analyses were performed with EZR (Saitama Medical Center, Jichi Medical University), which is a graphical user interface for R (The R Foundation for Statistical Computing, Vienna, Austria, version 3.61). It is a modified version of R Commander (version 1.41) designed to add statistical functions that are frequently used in biostatistics. We did a posthoc multiplicity adjustment with the Bonferroni method for the log‐rank tests.

## 3 RESULTS

### 3.1 Patient characteristics

In total, there were 511 patients with mRCC who received molecular targeted therapy. In the end, 398 patients with ccRCC and 67 patients with nccRCC who met the enrollment criteria were included in the analysis for determining the efficacy of systemic drug therapies in metastatic nccRCC (Figure 1). Table 1 shows the clinical characteristics of the mRCC patients at the start of fist‐line therapy administration. In the ccRCC group, 77.1% of the patients were male, and the mean age was 66 years (range, 37–87 years). In the nccRCC group, 65.7% of the patients were male, and the mean age was 63 years (range, 24–89 years). The nccRCC group was significantly younger (*p* = 0.008), and included significantly more females (*p* = 0.044) than the ccRCC group. The IMDC risk classification in the ccRCC and nccRCC groups was mostly intermediate (*n* = 174 and *n* = 31, respectively) or poor (*n* = 120 and *n* = 25, respectively; Table 1). The subtypes of nccRCC included papillary (*n* = 26), chromophobe (*n* = 6), collecting duct carcinoma (*n* = 6), unclassified (*n* = 16), microphthalmia‐associated transcription factor (Mit) family translocation RCC (*n* = 8), acquired cystic disease (ACD)‐related RCC (n = 4), mucinous tubular spindle cell carcinoma (*n* = 2), hereditary leiomyomatosis and renal cell cancer (HLRCC) (*n* = 1), and others (*n* = 8; Table 2). There was no difference in the IMDC risk among nccRCC subtypes.

**TABLE 1:** The patients characteristic of metastatic renal cell carcinoma who were administrated systemic drug therapy.

Patients characteristic	ccRCC		nccRCC		*p*‐Value
Cases	398		67		
Age (median ± SD)	66 ± 9.8	(37–87)	63 ± 14.2	(24–89)	0.008*
Sex					0.044*
Male	307 (77.1%)		44 (65.7%)		
Female	91 (22.9%)		23 (34.3%)		
Number of death	222		39		0.711
IMDC classification					0.529
Favorable	29		4		
Intermediate	174		31		
Poor	120		24		
Undetectable	75		8		
First‐line drug therapy					
Treatment period (day)	193 ± 480	(1–3625)	98 ± 422	(4–1875)	
First‐line drug					
Multitargeted tyrosine kinase inhibitors (TKIs)	391		60		
Sunitinib	221		28		
Sorafenib	36		9		
Axitinib	111		18		
Pazopanib	23		5		
Mammallian Target Of Rapamycin inhibitor (mTORI)	7		7		
Temsirolimus	4		4		
Everolimus	3		3		

* Indicated significance at *p* < 0.05.

**TABLE 2:** The pathology of renal cell carcinoma subtypes and IMDC classification.

Pathology of subtypes	Number of patients		IMDC classification			
			Favorable	Intermediate	Poor	Undetectable
Subtype (ISUP/WHO 2012)						
RCC	67		4	31	24	8
Papillary	23		3	12	7	1
Chromophobe	5		0	3	1	1
Collecting duct carcinoma	2		0	0	1	1
Unclassificated	14		0	3	9	2
MiT family translocation RCC	8		1	4	2	1
ACD related RCC	4		0	4	0	0
Mucinous tubular spindle cell carcinoma	2		0	1	0	1
HLRCC	1		0	1	0	0
Others	8		0	3	4	1

### 3.2 Comparison of the effects of molecular targeted therapies between ccRCC and nccRCC

**FIGURE 2:**
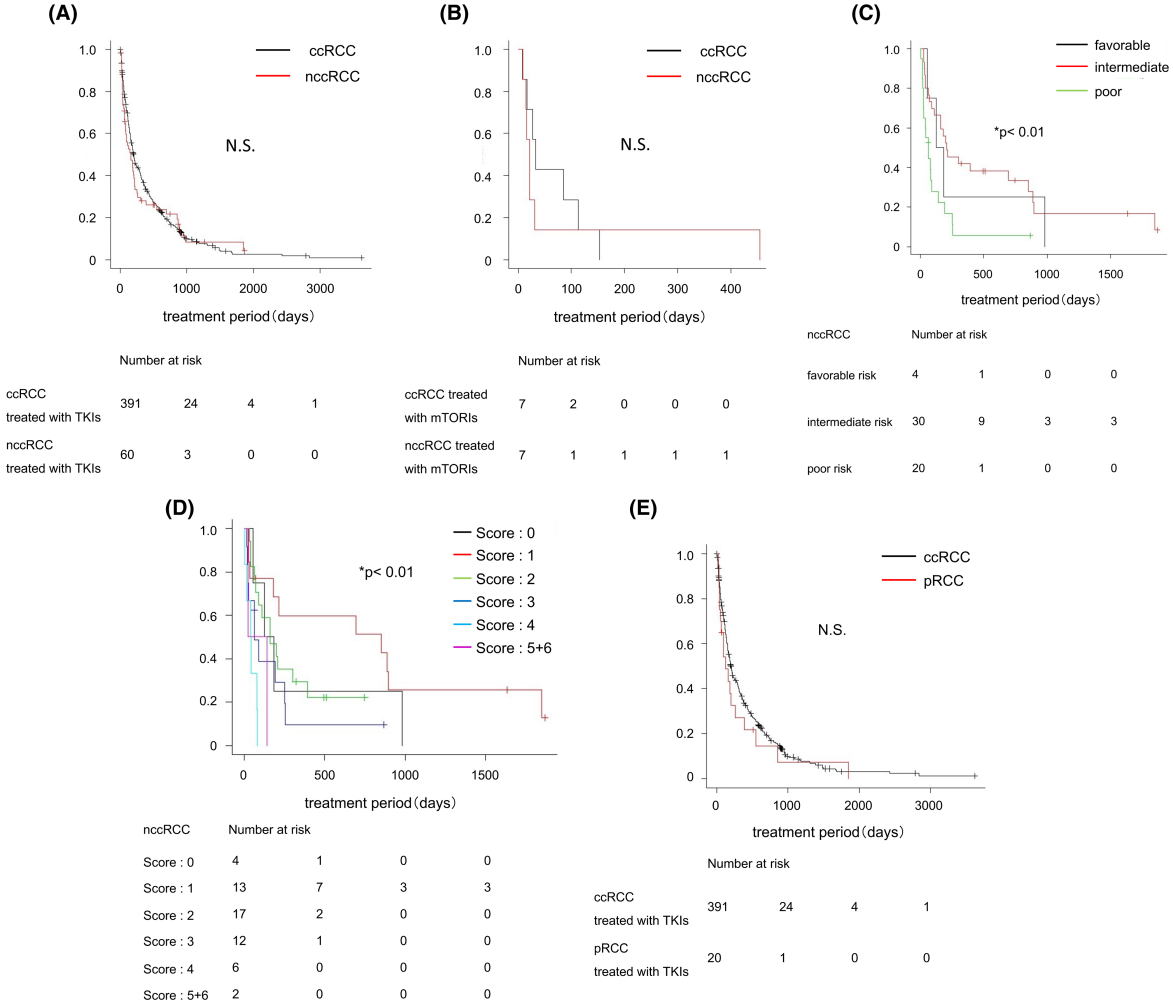
Comparison of the effects of molecular targeted therapy between ccRCC and nccRCC. (A) Kaplan–Meier estimate of the time to treatment failure (TTF) for multiple tyrosine kinase inhibitors (TKIs) as first‐line drug therapy. Comparison between clear cell renal cell carcinoma (ccRCC; black line) and non‐clear cell renal cell carcinoma (nccRCC; red line). (B) Kaplan–Meier estimate of the TTF for mammalian target of rapamycin inhibitor (mTORI) as first‐line drug therapy. Comparison between ccRCC (black line) and nccRCC (red line). (C) Kaplan–Meier estimate of the TTF for TKIs in nccRCC cases. Comparison between groups with a favorable risk (black line), intermediate risk (red line), and poor risk (green line) according to the IMDC risk classification before TKI administration. (D) Kaplan–Meier estimate of the TTF for TKIs in nccRCC cases. Comparison between groups with an IMDC risk score of 0 points (black line), 1 point (red line), 2 points (green line), 3 points (blue line), 4 points (light blue line), and 5 + 6 points (violet line) before TKI administration. (E) Kaplan–Meier estimate of the TTF for TKIs as first‐line drug therapy. Comparison between ccRCC (black line) and papillary renal cell carcinoma (pRCC; red line) groups. The *p*‐value was calculated by the log‐rank test, and was adjusted for using the Bonferroni method. N.S, not significant.

We compared the therapeutic effects of molecular targeted therapies between the ccRCC and nccRCC groups. There was no statistically significant difference in the IMDC risk classification between the ccRCC and nccRCC patients who received TKIs (Table 3) or mTORIs (Table 4) as first‐line therapy. Similarly, there was no difference in the IMDC risk score between the two groups (Tables 3 and 4). The TTF for TKIs as first‐line treatment was similar between the ccRCC (205 days, 95% confidence interval (CI): 174–243 days) and nccRCC (161 days, 95% CI: 75–212 days) groups (Figure 2A). There was no significant difference in the TTF for mTORIs as first‐line therapy between the ccRCC (33 days, 95% CI: 8–113 days) and nccRCC (21 days, 95% CI: 9–31 days) groups (Figure 2B). In contrast, in both the ccRCC (Figure S1a) and nccRCC (Figure 2C) groups, patients with a worse IMDC risk classification had a shorter TTF for TKIs as first‐line therapy (*p* < 0.01). ccRCC and nccRCC patients with a worse IMDC risk score also had a shorter TTF for TKIs as first‐line therapy (*p* < 0.01; Figure 2D, Figure S1b).

**TABLE 3:** The patients characteristic of metastatic renal cell carcinoma who were administrated the first line treatment with TKIs.

Patients characteristic	ccRCC		nccRCC		*p*‐Value
Cases	391		60		
Age (median ± SD)	67 ± 9.7	(37–87)	63 ± 14.7	(24–89	0.002*
Sex					0.020*
Male	302 (77.2%)		38 (63.3%)		
Female	89 (22.8%)		22 (36.7%)		
IMDC classification					0.812
Favorable	29		4		
Intermediate	173		30		
Poor	115		20		
Undetectable	74		6		
IMDC risk score					0.942
0	29		4		
1	68		13		
2	105		17		
3	70		12		
4	31		6		
5	10		1		
6	4		1		
Undetectable	74		6		

* Indicated significance at *p* < 0.05.

**TABLE 4:** : The patients characteristic of metastatic renal cell carcinoma who were administrated the first line treatment with mTORIs.

Patients characteristic	ccRCC		nccRCC		*p*‐Value
Cases	7		7		
Age (median ± SD)	63 ± 12.4	(47–78)	68 ± 10.3	(55–82)	0.442
Sex					0.591
Male	2 (28.6%)		1 (14.3%)		
Female	5 (71.4%)		6 (85.7%)		
IMDC classification					1
Favorable	0		0		
Intermediate	1		1		
Poor	5		4		
Undetectable	1		2		
IMDC risk score					0.636
0	0		0		
1	0		0		
2	1		1		
3	1		2		
4	3		1		
5	0		1		
6	1		0		
Undetectable	1		2		

* Indicated significance at *p* < 0.05.

As further analysis, we compared the therapeutic effects of TKIs between ccRCC and papillary RCC (pRCC), which was a major pathological subtype in nccRCC. There was no significant difference in the IMDC risk classification between the ccRCC and pRCC patients who received TKIs as first‐line therapy (Table 5). The exact scores of IMDC risk between the two groups were not different (Table 5). The TTF for TKIs as first‐line treatment was similar between the ccRCC (205 days, 95% CI: 174–243 days) and pRCC (127 days, 95% CI: 41–258 days) groups (Figure 2E).

**TABLE 5:** The patients characteristic of metastatic clear cell renal cell carcinoma and papillary renal cell carcinoma who were administrated the first line treatment with TKIs.

Patients characteristic	ccRCC		pRCC		*p*‐Value
Cases	391		20		
Age (median ± SD)	67 ± 9.7	(37–87)	64.5 ± 17.5	(29–89)	0.399
Sex					0.208
Male	302 (77.2%)		7 (35.0%)		
Female	89 (22.8%)		13 (65.0%)		
IMDC classification					0.274
Favorable	29		3		
Intermediate	173		11		
Poor	115		5		
Undetectable	74		1		
IMDC risk score					0.153
0	29		3		
1	68		5		
2	105		6		
3	70		5		
4	31		0		
5	10		0		
6	4		0		
Undetectable	74		1		

* Indicated significance at *p* < 0.05.

### 3.3 Comparison of the effects of various agents as molecular targeted therapy for nccRCC

**FIGURE 3:**
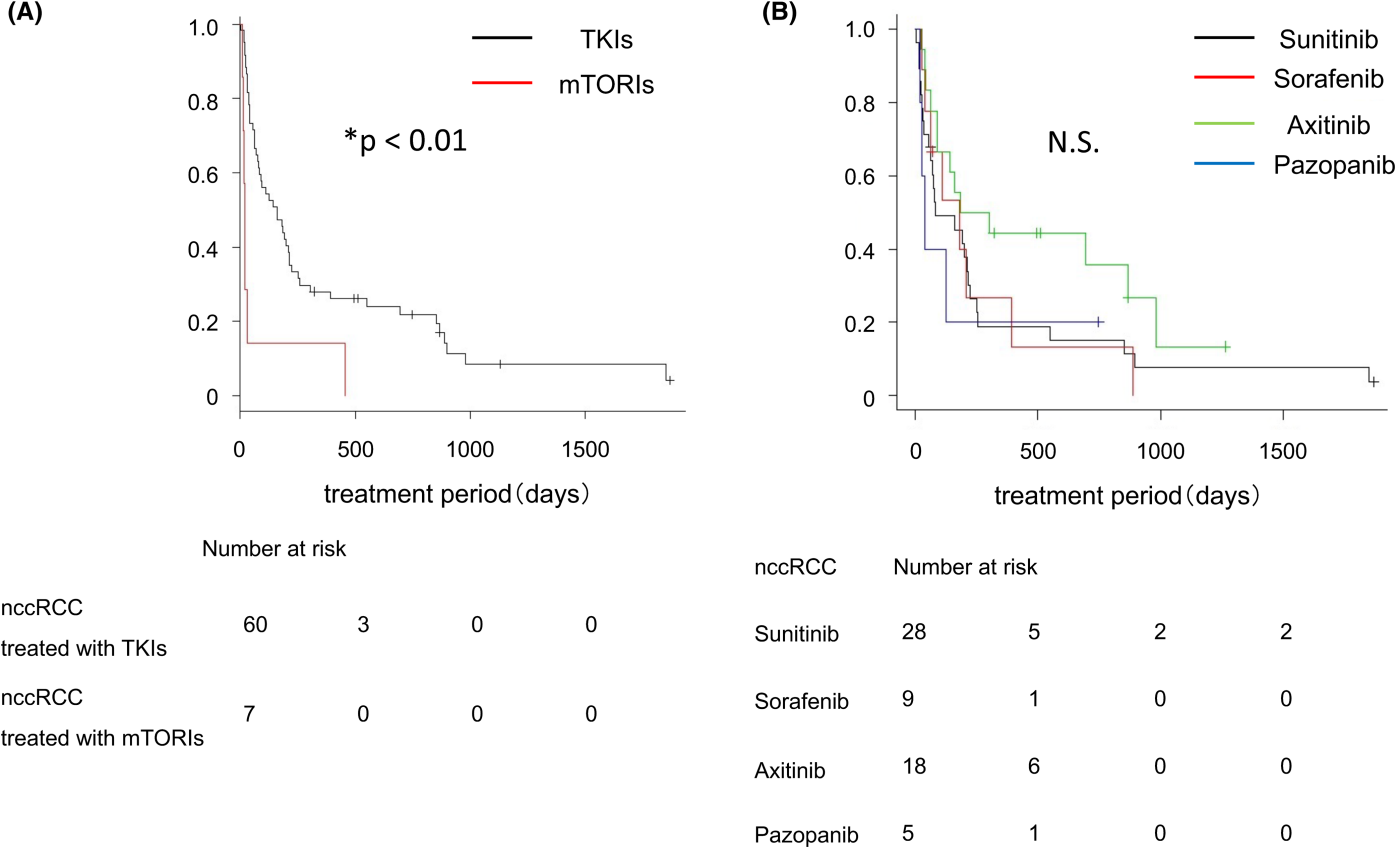
Comparison of the effects of various agents as molecular targeted therapy for nccRCC. (A) Kaplan–Meier estimate of the time to treatment failure (TTF) for tyrosine kinase inhibitors (TKIs) and mammalian target of rapamycin inhibitor (mTORI) as first‐line drug therapy. Comparison between TKIs (black line) and mTORI (red line). (B) Kaplan–Meier estimate of the TTF for sunitinib, sorafenib, axitinib, and pazopanib as first‐line drug therapy. Comparison between sunitinib (black line), sorafenib (red line), axitinib (green line), and pazopanib (blue line). The *p*‐value was calculated by the log‐rank test, and was adjusted for using the Bonferroni method. N.S, not significant.

There was no statistically significant difference in the IMDC risk classification between nccRCC patients treated with TKIs or mTORIs. We then compared the therapeutic effects of TKIs and mTORIs for nccRCC. The TTF for TKIs as first‐line therapy (161 days, 95% CI: 75–212 days) was significantly longer than that for mTORIs (21 days, 95% CI: 9–31 days, *p* < 0.01; Figure 3A). Additionally, we examined whether there was a difference in the efficacy between the TKIs. Although there was no significant difference in the TTF between the TKIs (sunitinib, 85 days, 95% CI: 56–215 days; sorafenib, 183 days, 95% CI: 28–393 days; pazopanib, 41 days, 95% CI: 21 days to not applicable (NA); and axitinib, 244 days, 95% CI: 90–981 days), the TTF for axitinib tended to be longer (Figure 3B).

### 3.4 Comparison of the effects of nivolumab for ccRCC and nccRCC

**FIGURE 4:**
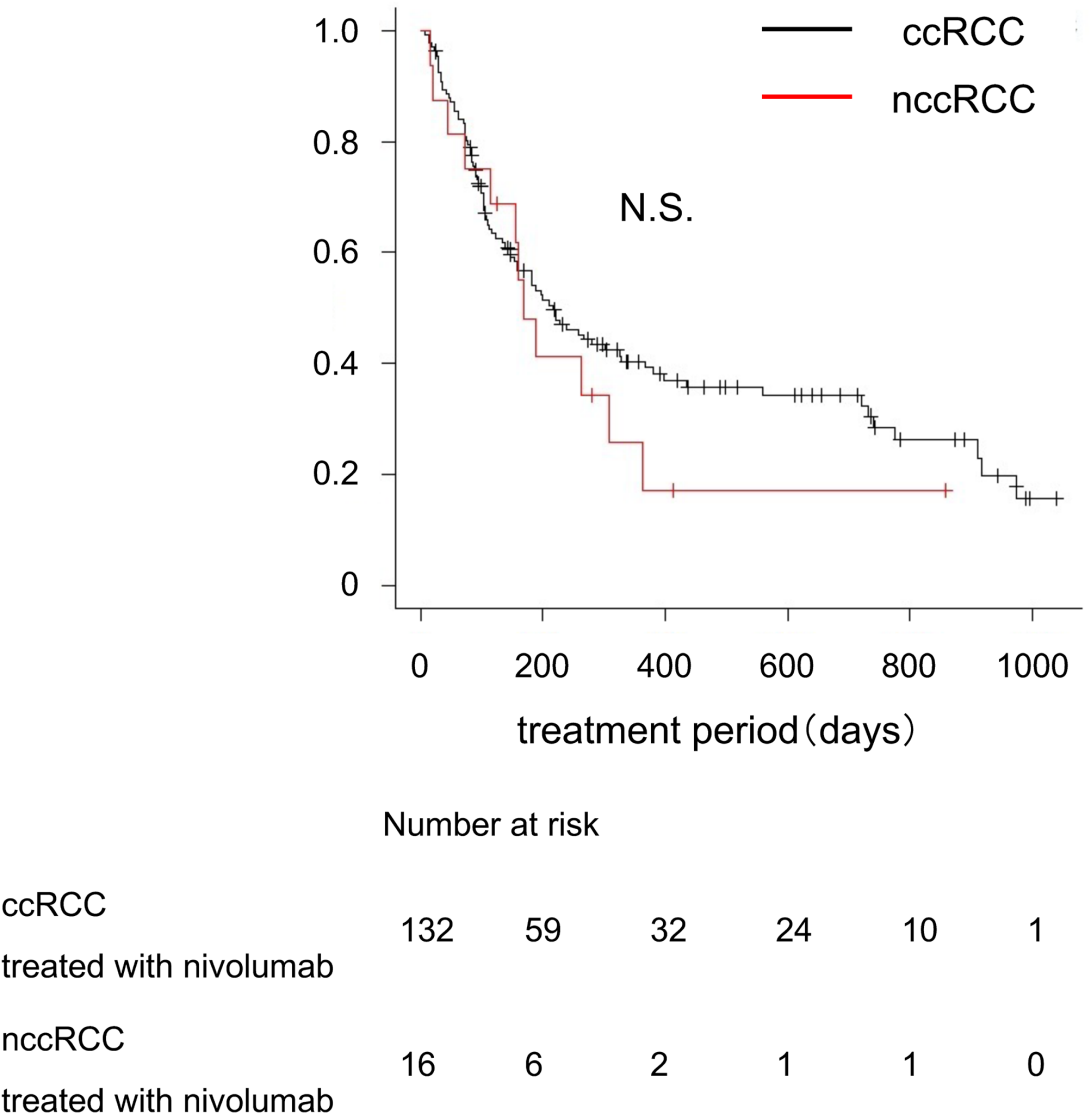
Comparison of the effects of nivolumab for ccRCC and nccRCC. Kaplan–Meier estimate of the time to treatment failure (TTF) for nivolumab therapy. Comparison between clear cell renal cell carcinoma (ccRCC; black line) and non‐clear cell renal cell carcinoma (nccRCC; red line) groups. The p‐value was calculated by the log‐rank test. N.S, not significant.

In total, 132 patients with ccRCC and 16 patients with nccRCC received nivolumab after prior therapy with systemic molecular targeted therapies. Comparisons of the background patient characteristics revealed a significant difference in age, but not in the IMDC risk classification between the ccRCC and nccRCC groups (Table 6). The exact scores of IMDC risk between the two groups were not different (Table 6). There was no statistically significant difference in the TTF for nivolumab between the ccRCC (217 days, 95% CI: 154–329 days) and nccRCC (168 days, 95% CI: 73–308 days) groups (Figure 4).

**TABLE 6:** : The patients characteristic of metastatic renal cell carcinoma who were administrated a nivolumab.

Patients characteristic	ccRCC		nccRCC		*p*‐Value
Cases	132		16		
Age (median ± SD)	67 ± 8.9	(41–83)	64 ± 11.6	(30–73)	0.030*
Sex					0.453
Male	102 (77.3%)		11 (68.8%)		
Female	30 (22.7%)		5 (31.2%)		
IMDC classification					0.282
Favorable	22		1		
Intermediate	58		7		
Poor	25		4		
Undetectable	27		4		
IMDC risk score					0.129
0	22		1		
1	31		2		
2	27		5		
3	16		2		
4	5		1		
5	2		1		
6	2		0		
Undetectable	27		4		
Pathology of subtypes					
Papillary			6		
Chromophobe			0		
Collecting duct carcinoma			1		
Unclassificated			3		
MiT family translocation RCC			3		
ACD related RCC			0		
Mucinous tubular spindle cell carcinoma			2		
HLRCC			0		
Others			1		

* Indicated significance at *p* < 0.05.

**FIGURE 5:**
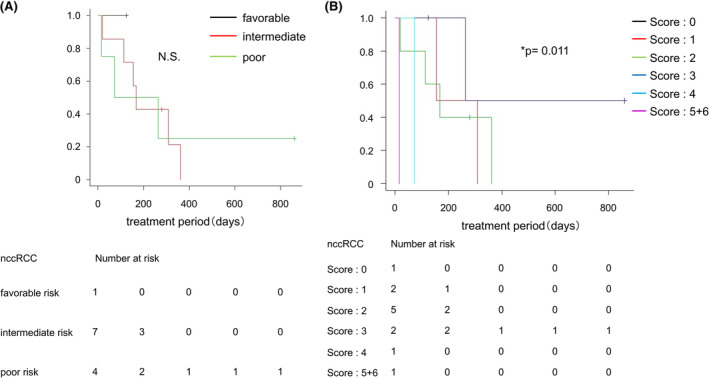
Comparison of the effects of nivolumab on the IMDC risk classification and IMDC risk score in ccRCC and nccRCC. (A) Kaplan–Meier estimate of the TTF for nivolumab therapy in non‐clear cell renal cell carcinoma (nccRCC) cases. Comparison between groups with a favorable risk (black line), intermediate risk (red line), and poor risk (green line) according to the IMDC risk classification before nivolumab administration. (B) Kaplan–Meier estimate of the TTF for nivolumab therapy in nccRCC cases. Comparison between groups with an IMDC risk score of 0 points (black line), 1 point (red line), 2 points (green line), 3 points (blue line), 4 points (light blue line), and 5 + 6 points (violet line) before nivolumab administration. The p‐value was calculated by the log‐rank test, and was adjusted for using the Bonferroni method. N.S, not significant.

In both the ccRCC and nccRCC patients treated with nivolumab, there was no difference in the TTF for nivolumab among the IMDC risk classification groups (Figure 5A and Figure S2a). On the other hand, a significant difference in the TTF for nivolumab was found among the IMDC risk score groups in nccRCC, although this difference was not found in ccRCC (Figure 5B and Figure S2b).

## 4 DISCUSSION

Although most previous clinical studies of systemic drug therapies for mRCC have focused on ccRCC,^5^, ^11^, ^12^, ^16^, ^17^, ^18^ there have been a few clinical pharmacotherapy studies related to nccRCC.^19^, ^20^, ^21^, ^22^ Vera‐Badillo et al. reported that patients with nccRCC who used molecular targeted agents had significantly lower response rates and shorter progression‐free survival (PFS) and overall survival than those with ccRCC.^19^ An analysis of a randomized phase II trial (RECORD‐3) performed by Motzer et al. showed that the PFS in patients treated with everolimus and sunitinib was shorter in nccRCC cases than in ccRCC cases.^20^ Another study reported that metastatic nccRCC was characterized by resistance to systemic therapy and poor survival.^21^ Ravaud et al. performed a prospective phase II study, and reported that sunitinib showed efficacy in treating types 1 and 2 pRCC, but the efficacy was lower than that in ccRCC.^22^ These findings indicate that the therapeutic effects, that is, the effects on overall survival, PFS, and tumor reduction, of TKIs and mTORIs are inferior in nccRCC than in ccRCC.^19^, ^20^, ^21^, ^22^ On the other hand, a phase II study performed by Jung et al. demonstrated that TKIs had promising effects and a tolerable safety profile in patients with metastatic nccRCC.^23^ Similarly, our retrospective analysis of Japanese patients with RCC revealed that the therapeutic effects of TKIs and mTORIs in nccRCC did not differ significantly from those in ccRCC. In addition, although there was no statically significant difference in the response to first‐generation TKIs (sunitinib, sorafenib, and pazopanib) and a second‐generation TKI (axitinib) in this study, the TTF for axitinib tended to be longer than that for the other TKIs. Although a small number of phase II trials of systemic drug therapies, including axitinib for nccRCC, have recently been conducted, more clinical data are needed, especially on axitinib.^24^, ^25^ Park et al. reported that axitinib showed promising efficacy, in terms of the objective response rate (ORR) and PFS, in recurrent or metastatic nccRCC when used after temsirolimus treatment has failed.^24^ Negrier et al. reported that axitinib had some efficacy in metastatic pRCC patients, especially in type 2 pRCC.^25^ Therefore, the choice of axitinib as a second‐generation TKI might affect the effectiveness of treatments and the treatment outcomes in patients with nccRCC in Japan.

In the present study, we also analyzed the efficacy of nivolumab after first‐line therapy. The results showed that there was no significant difference in the TTF for nivolumab in ccRCC and nccRCC patients, indicating that nivolumab is useful as drug therapy for nccRCC as well as for ccRCC. In ccRCC patients, there was no difference in the therapeutic effects of nivolumab according to the IMDC risk classification and IMDC risk score. In contrast, in nccRCC patients, the TTF for nivolumab became shorter with increasing IMDC risk score. Although this result might be due to the small number of nccRCC cases that received nivolumab in our study, it is possible that the risk score might affect the effectiveness of nivolumab. Previous reports have demonstrated the therapeutic efficacy of IO therapy for nccRCC, but the numbers of cases in those studies were also limited.^13^, ^26^, ^27^ Therefore, there is currently insufficient data for accurately evaluating the effect of nivolumab in nccRCC and in each histological type. Further studies are needed to evaluate the long‐term survival of nccRCC patients treated with nivolumab.

Our analysis revealed that the efficacy of TKIs and nivolumab for nccRCC was comparable to that for ccRCC. The recommendations for systemic therapy in metastatic renal cancer have recently been undergoing dramatic changes in Japan, and the recommended first‐line therapy for ccRCC has become IO combination therapy or IO plus VEGFR‐TKI therapy. Therefore, it appears that combination therapies are the mainstay of systemic therapies for nccRCC.^28^ Our analysis results suggested that nccRCC patients with a high risk score might respond poorly to nivolumab. Accordingly, these patients are possible to benefit from IO plus VEGFR‐TKI therapy such as axitinib combination therapy. Since it is difficult to conduct large‐scale clinical trials on nccRCC due to the small number of cases, more data accumulation on nccRCC cases in central cancer centers, and clinical research collaborations between more institutions are needed.

There are several limitations in this study. First is the retrospective study design and the small number of nccRCC cases, which prevented us from analyzing each histological type individually. In addition, the majority of our patients had pRCC. Previous studies have reported that TKIs were effective for metastatic pRCC.^25^

Second, the recommended therapies for mRCC changed dramatically during the case collection period. Therefore, it is possible that this was due to the changes in the available drugs at different times. For example, the current first‐line therapies for mRCC are immune combination therapies, but at the time of our study, mRCC cases could not be treated with nivolumab as first‐line therapy in Japan due to the regulations of the national medical insurance system (Each drug has been available in Japan; sorafenib since January 2008, sunitinib since June 2008, axitinib since August 2012, pazopanib since March 2014, and nivolumab since September 2016.). The results of our study may not necessarily be applicable to clinical practice, as the recommended therapies for mRCC in the past differ greatly from the current recommendations. However, the results of this study might still be useful for drug selection in combination therapies for mRCC.

## 5 CONCLUSIONS

The present study showed that the therapeutic efficacy of TKIs and mTORIs as first‐line therapy for nccRCC was similar to that for ccRCC in Japanese patients. Furthermore, our analysis indicated that TKIs were effective as first‐line therapy for nccRCC. The results also indicated that the effect of nivolumab on nccRCC was comparable to that on ccRCC. In future studies, a larger number of cases is needed to evaluate the therapeutic effects on each histological type of nccRCC, and the response to novel combination therapies.

## AUTHOR CONTRIBUTIONS

**Tomoyuki Koguchi:** Investigation (equal); writing – original draft (equal). **Sei Naito:** Resources (equal). **Shingo Hatakeyama:** Resources (equal). **Kazuyuki Numakura:** Resources (equal). **Yumina Muto:** Resources (equal). **Renpei Kato:** Resources (equal). **Takahiro Kojima:** Resources (equal). **Yoshihide Kawasaki:** Resources (equal). **Kento Morozumi:** Resources (equal). **Shuya Kandori:** Resources (equal). **Sadafumi Kawamura:** Resources (equal). **Hiroyuki Nishiyama:** Conceptualization (supporting). **Akihiro Ito:** Conceptualization (supporting). **Tomonori Habuchi:** Conceptualization (supporting). **Wataru Obara:** Conceptualization (supporting). **Chikara Ohyama:** Conceptualization (supporting). **Norihiko Tsuchiya:** Conceptualization (supporting). **Yoshiyuki Kojima:** Supervision (equal).

## FUNDING INFORMATION

No funding was provided for research design, data collection and analysis, interpretation of data, or writing of the manuscript.

## CONFLICT OF INTEREST STATEMENT

The authors have no conflict of interest.

## Supporting information


Figure S1.

Figure S2.

## Data Availability

Data sharing is not applicable to this article as no new data were created or analyzed in this study.

## References

[1] Mai KT , Faraji H , Desantis D , Robertson SJ , Belanger EC , Levac J . Renal cell carcinoma with mixed features of papillary and clear cell cytomorphology: a fluorescent in situ hybridization study. Virchows Arch. 2010;456:77‐84. 20033232 10.1007/s00428-009-0871-2

[2] Sims JN , Yedjou CG , Abugri D , et al. Racial disparities and preventive measures to renal cell carcinoma. Int J Environ Res Public Health. 2018;15:15. 10.3390/ijerph15061089 PMC6024978 29843394

[3] Albiges L , Molinie V , Escudier B . Non‐clear cell renal cell carcinoma: does the mammalian target of rapamycin represent a rational therapeutic target? Oncologist. 2012;17:1051‐1062. 22807514 10.1634/theoncologist.2012-0038 PMC3425523

[4] <10815_2015_article_468.Pdf>.

[5] Bedke J , Gauler T , Grünwald V , et al. Systemic therapy in metastatic renal cell carcinoma. World J Urol. 2017;35:179‐188. 27277600 10.1007/s00345-016-1868-5 PMC5272893

[6] Armstrong AJ , Halabi S , Eisen T , et al. Everolimus versus sunitinib for patients with metastatic non‐clear cell renal cell carcinoma (aspen): a multicentre, open‐label, randomised phase 2 trial. Lancet Oncol. 2016;17:378‐388. 26794930 10.1016/S1470-2045(15)00515-X PMC6863151

[7] Tannir NM , Jonasch E , Albiges L , et al. Everolimus versus sunitinib prospective evaluation in metastatic non‐clear cell renal cell carcinoma (espn): a randomized multicenter phase 2 trial. Eur Urol. 2016;69:866‐874. 26626617 10.1016/j.eururo.2015.10.049 PMC4879109

[8] Dutcher JP , de Souza P , McDermott D , et al. Effect of temsirolimus versus interferon‐alpha on outcome of patients with advanced renal cell carcinoma of different tumor histologies. Medical Oncol. 2009;26:202‐209. 10.1007/s12032-009-9177-0 19229667

[9] Bergmann L , Grünwald V , Maute L , et al. A randomized phase iia trial with temsirolimus versus sunitinib in advanced non‐clear cell renal cell carcinoma: an intergroup study of the Cesar central european society for anticancer drug research‐ewiv and the interdisciplinary working group on renal cell cancer (iagn) of the german cancer society. Oncology Res Treat. 2020;43:333‐339. 10.1159/000508450 32541143

[10] Wallis CJD , Butaney M , Satkunasivam R , et al. Association of patient sex with efficacy of immune checkpoint inhibitors and overall survival in advanced cancers: a systematic review and meta‐analysis. JAMA Oncol. 2019;5:529‐536. 30605213 10.1001/jamaoncol.2018.5904 PMC6459215

[11] Motzer RJ , Escudier B , McDermott DF , et al. Nivolumab versus everolimus in advanced renal‐cell carcinoma. N Engl J Med. 2015;373:1803‐1813. 26406148 10.1056/NEJMoa1510665 PMC5719487

[12] Tomita Y , Fukasawa S , Shinohara N , et al. Nivolumab versus everolimus in advanced renal cell carcinoma: Japanese subgroup 3‐year follow‐up analysis from the phase iii checkmate 025 study. Jpn J Clin Oncol. 2019;49:506‐514. 30941424 10.1093/jjco/hyz026

[13] Chahoud J , Msaouel P , Campbell MT , et al. Nivolumab for the treatment of patients with metastatic non‐clear cell renal cell carcinoma (nccrcc): a single‐institutional experience and literature meta‐analysis. Oncologist. 2019;25:252‐258. 32162795 10.1634/theoncologist.2019-0372 PMC7066696

[14] Heng DY , Xie W , Regan MM , et al. External validation and comparison with other models of the international metastatic renal‐cell carcinoma database consortium prognostic model: a population‐based study. Lancet Oncol. 2013;14:141‐148. 23312463 10.1016/S1470-2045(12)70559-4 PMC4144042

[15] Gajra A , Zemla TJ , Jatoi A , et al. Time‐to‐treatment‐failure and related outcomes among 1000+ advanced non‐small cell lung cancer patients: comparisons between older versus younger patients (alliance a151711). J Thorac Oncol. 2018;13:996‐1003. 29608967 10.1016/j.jtho.2018.03.020 PMC6015776

[16] Makhov P , Joshi S , Ghatalia P , Kutikov A , Uzzo RG , Kolenko VM . Resistance to systemic therapies in clear cell renal cell carcinoma: mechanisms and management strategies. Mol Cancer Ther. 2018;17:1355‐1364. 29967214 10.1158/1535-7163.MCT-17-1299 PMC6034114

[17] Atkins MB , Tannir NM . Current and emerging therapies for first‐line treatment of metastatic clear cell renal cell carcinoma. Cancer Treat Rev. 2018;70:127‐137. 30173085 10.1016/j.ctrv.2018.07.009

[18] Shah AY , Kotecha RR , Lemke EA , et al. Outcomes of patients with metastatic clear‐cell renal cell carcinoma treated with second‐line vegfr‐tki after first‐line immune checkpoint inhibitors. Europ J Canc. 1990;2019(114):67‐75. 10.1016/j.ejca.2019.04.003 PMC7537491 31075726

[19] Vera‐Badillo FE , Templeton AJ , Duran I , et al. Systemic therapy for non‐clear cell renal cell carcinomas: a systematic review and meta‐analysis. Eur Urol. 2015;67:740‐749. 24882670 10.1016/j.eururo.2014.05.010

[20] Motzer RJ , Barrios CH , Kim TM , et al. Phase ii randomized trial comparing sequential first‐line everolimus and second‐line sunitinib versus first‐line sunitinib and second‐line everolimus in patients with metastatic renal cell carcinoma. J Clin Oncol. 2014;32:2765‐2772. 25049330 10.1200/JCO.2013.54.6911 PMC5569681

[21] Motzer RJ , Bacik J , Mariani T , Russo P , Mazumdar M , Reuter V . Treatment outcome and survival associated with metastatic renal cell carcinoma of non‐clear‐cell histology. J Clin Oncol. 2002;20:2376‐2381. 11981011 10.1200/JCO.2002.11.123

[22] Ravaud A , Oudard S , De Fromont M , et al. First‐line treatment with sunitinib for type 1 and type 2 locally advanced or metastatic papillary renal cell carcinoma: a phase ii study (supap) by the french genitourinary group (getug)†. Annal Oncol. 2015;26:1123‐1128. 10.1093/annonc/mdv149 25802238

[23] Jung KS , Lee SJ , Park SH , et al. Pazopanib for the treatment of non‐clear cell renal cell carcinoma: a single‐arm, open‐label, multicenter, phase ii study. Canc Res Treat. 2018;50:488‐494. 10.4143/crt.2016.584 PMC5912146 28546525

[24] Park I , Lee SH , Lee JL . A multicenter phase ii trial of axitinib in patients with recurrent or metastatic non‐clear‐cell renal cell carcinoma who had failed prior treatment with temsirolimus. Clin Genitourin Cancer. 2018;16:e997‐e1002. 29903415 10.1016/j.clgc.2018.05.011

[25] Negrier S , Rioux‐Leclercq N , Ferlay C , et al. Axitinib in first‐line for patients with metastatic papillary renal cell carcinoma: results of the multicentre, open‐label, single‐arm, phase ii axipap trial. Europ J Canc. 2020;129:107‐116. 10.1016/j.ejca.2020.02.001 32146304

[26] Hinata N , Yonese J , Masui S , et al. A multicenter retrospective study of nivolumab monotherapy in previously treated metastatic renal cell carcinoma patients: interim analysis of japanese real‐world data. Int J Clin Oncol. 2020;25:1533‐1542. 32519026 10.1007/s10147-020-01692-z PMC7392942

[27] Vogelzang NJ , Olsen MR , McFarlane JJ , et al. Safety and efficacy of nivolumab in patients with advanced non‐clear cell renal cell carcinoma: results from the phase iiib/iv checkmate 374 study. Clin Genitourin Cancer. 2020;18:461‐468.e3. 32718906 10.1016/j.clgc.2020.05.006

[28] Gupta R , Ornstein MC , Li H , et al. Clinical activity of ipilimumab plus nivolumab in patients with metastatic non‐clear cell renal cell carcinoma. Clin Genitourin Cancer. 2020;18:429‐435. 32800717 10.1016/j.clgc.2019.11.012

