# Supplementary figures and images for: The efficacy of molecular targeted therapy and nivolumab therapy for metastatic non‐clear cell renal cell carcinoma: A retrospective analysis using the Michinoku Japan urological cancer study group database
Source: Cancer Med. 2023 Oct 31;12(22):20677–89. doi: 10.1002/cam4.6591 (PMC10709721; doi:10.1002/cam4.6591)

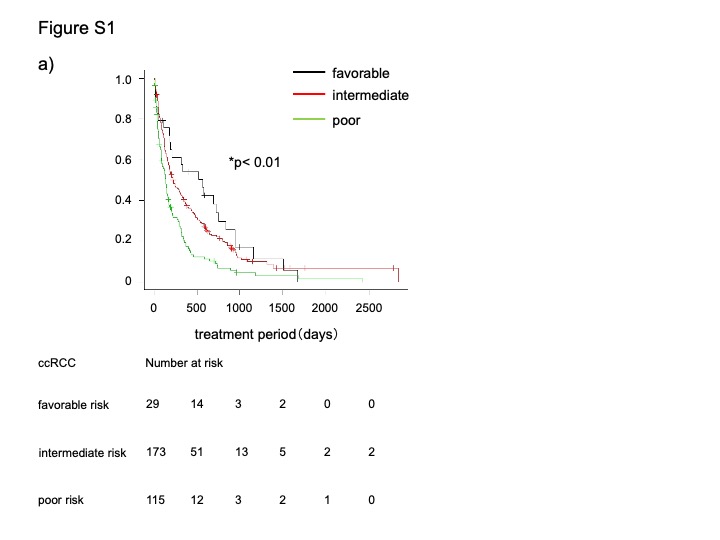

Supplement: Supplementary file 1 — Figure S1. [file CAM4-12-20677-s001.zip › figure S1a.jpeg]

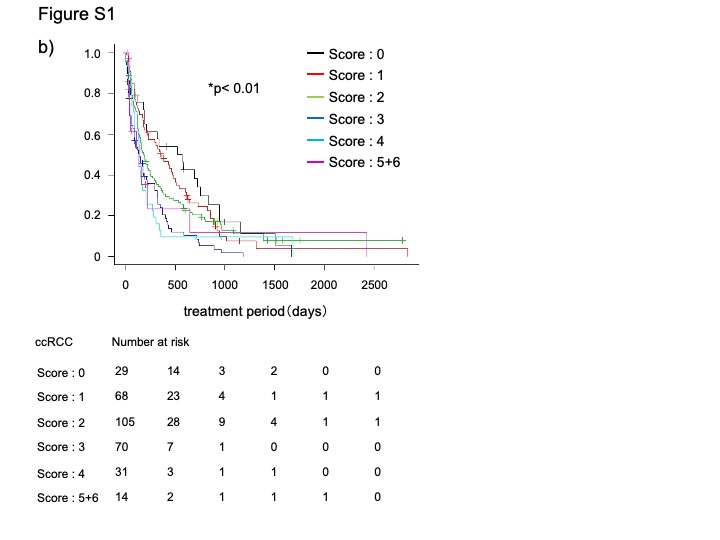

Supplement: Supplementary file 1 — Figure S1. [file CAM4-12-20677-s001.zip › figure S1b.jpeg]

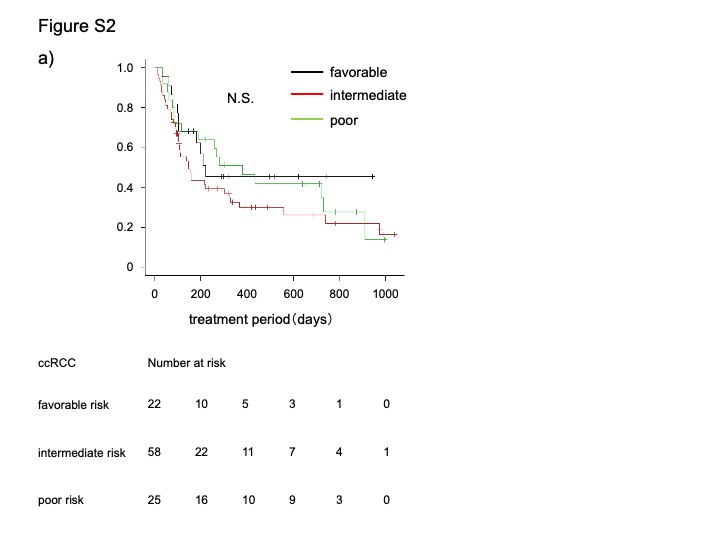

Supplement: Supplementary file 2 — Figure S2. [file CAM4-12-20677-s002.zip › figure S2a.jpeg]

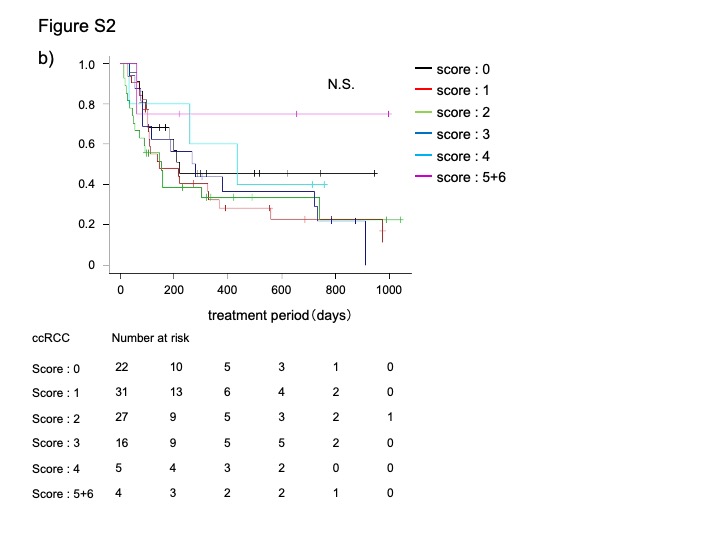

Supplement: Supplementary file 2 — Figure S2. [file CAM4-12-20677-s002.zip › figure S2b.jpeg]
